# Regulation of Stromal Cells by Sex Steroid Hormones in the Breast Cancer Microenvironment

**DOI:** 10.3390/cancers16234043

**Published:** 2024-12-02

**Authors:** Mio Yamaguchi-Tanaka, Kiyoshi Takagi, Ai Sato, Yuto Yamazaki, Minoru Miyashita, Atsushi Masamune, Takashi Suzuki

**Affiliations:** 1 Personalized Medicine Center, Tohoku University Hospital, Sendai 980-8574, Japan; 2 Department of Pathology and Histotechnology, Tohoku University Graduate School of Medicine, Sendai 980-8575, Japantakashi.suzuki.c1@tohoku.ac.jp (T.S.); 3 Department of Pathology, Tohoku University Hospital, Sendai 980-8574, Japan; 4 Department of Breast and Endocrine Surgical Oncology, Tohoku University Graduate School of Medicine, Sendai 980-8574, Japan; 5 Division of Gastroenterology, Tohoku University Graduate School of Medicine, Sendai 980-8574, Japan; 6 Department of Anatomic Pathology, Tohoku University Graduate School of Medicine, Sendai 980-8575, Japan

**Keywords:** breast cancer, tumor microenvironment, estrogen, androgen

## Abstract

Breast cancer is a prevalent hormone-dependent malignancy, and estrogens/estrogen receptor (ER) signaling are pivotal therapeutic targets in ER-positive breast cancers, where endocrine therapy has significantly improved treatment efficacy. However, the emergence of both de novo and acquired resistance to these therapies continues to pose challenges. Additionally, androgens are produced locally in breast carcinoma tissues by androgen-producing enzymes, and the androgen receptor (AR) is commonly expressed in breast cancer cells. Intratumoral androgens play a significant role in breast cancer progression and are closely linked to resistance to endocrine treatments. The tumor microenvironment, consisting of tumor cells, immune cells, fibroblasts, extracellular matrix, and blood vessels, is crucial for tumor progression. Stromal cells influence tumor progression through direct interactions with cancer cells, the secretion of soluble factors, and modulation of tumor immunity. Estrogen and androgen signaling in breast cancer cells affects the tumor microenvironment, and the expression of hormone receptors correlates with the diversity of the stromal cell profile. Notably, various stromal cells also express ER or AR, which impacts breast cancer development. This review describes how sex steroid hormones, particularly estrogens and androgens, affect stromal cells in the breast cancer microenvironment. We summarize recent findings focusing on the effects of ER/AR signaling in breast cancer cells on stromal cells, as well as the direct effects of ER/AR signaling in stromal cells.

## Simple Summary

Sex steroid hormones, including estrogens and androgens, are synthesized locally in breast cancer tissue. The estrogen/estrogen receptor (ER) signaling pathway serves as a major therapeutic target for endocrine therapy in ER-positive breast cancer; however, resistance to these therapies poses significant challenges. The androgen receptor (AR) is also highly expressed in breast cancer cells, and intratumoral androgens are implicated in tumor progression and resistance to endocrine therapy. The tumor microenvironment comprises not only tumor cells but also various stromal cells, which play a critical role in tumor development. Estrogen and androgen signaling influence the tumor microenvironment, and variations in hormone receptor expression in breast cancer lead to stromal cell diversity. This review focuses on the roles of ER and AR signaling in both breast cancer cells and stromal cells, summarizing recent findings regarding the effects of estrogens and androgens on stromal cells within the breast cancer microenvironment.

## 1. Introduction

Breast cancer is a classic example of a hormone-dependent malignancy and stands as the most prevalent cancer among women globally. Active steroids, including estrogens and androgens, are synthesized locally within breast cancer tissues by enzymes responsible for sex steroid production [1]. Specifically, the signaling pathways of estrogen and its receptor (ER) serve as critical therapeutic targets in ER-positive breast cancers, and endocrine therapy has significantly enhanced the effectiveness of treatment for this condition. Nevertheless, both de novo and acquired resistance to endocrine therapy frequently occur, posing a significant challenge that needs to be addressed. In addition, androgen-producing enzymes are commonly found in breast carcinoma tissues, aiding in the synthesis of intratumoral androgens [2]. Androgen receptor (AR) is also frequently present in breast cancer cells, and intratumoral androgens are important for the progression of breast cancers while also being closely associated with resistance to endocrine therapies in breast cancers [2].

To date, there has been extensive research into new therapeutic targets and biomarkers in breast cancer cells [3,4,5,6]. On the other hand, the breast cancer microenvironment is composed not only of cancer cells but also of various components such as immune cells, fibroblasts, extracellular matrix, adipose tissues, and blood vessels, where the interactions between tumor cells and these components are closely linked to tumor progression [7,8,9,10,11,12]. Stromal cells facilitate tumor progression through direct interactions with cancer cells and the secretion of a variety of proteins, including cytokines, which regulate tumor immunity and angiogenesis. Previous research has indicated that sex steroid hormones and their receptor signaling pathways in breast cancer cells influence the tumor microenvironment, and that hormone receptor expression status is closely associated with the diversity of the immune cell profile [13]. Additionally, various stromal cells express either ER or AR, contributing to the development of breast cancer [14]. This review aims to summarize recent findings on the effects of sex steroid hormones, particularly estrogens and androgens, on the breast cancer microenvironment. It focuses on the influence of sex steroid hormones and their receptor signaling in breast cancer cells on stromal cells (indirect effects), as well as the actions of sex steroid hormones via their receptors in stromal cells (direct effects).

## 2. Estrogens and Androgens in Breast Cancer

### 2.1. Intratumoral Synthesis of Estrogens and Androgens

In premenopausal women, circulating estrogens are primarily secreted by the ovaries. After menopause, however, estrogen synthesis predominantly occurs in peripheral tissues, including breast tissue, through the conversion of circulating inactive steroids such as androstenedione and estrone sulfate [15]. Enzymes involved in estrogen synthesis, such as aromatase, steroid sulfatase (STS), and 17β-hydroxysteroid dehydrogenase type 1 (17βHSD1) [16,17,18,19,20], as well as androgen synthesis enzymes 17βHSD5 and 5α-reductase type 1 (5αRed1) [1,2,21,22,23], are expressed in several malignant tissues, including breast, lung, and endometrial cancers, and biologically active estrogens and androgens, estradiol (E2) and dihydrotestosterone (DHT), are locally synthesized [24,25,26]. Aromatase, in particular, is a key enzyme in estrogen synthesis, primarily catalyzing the aromatization of androstenedione and testosterone to estrogens, and serves as an important therapeutic target for endocrine therapy using aromatase inhibitors (AI) in breast cancer patients [15]. Therefore, aromatase negatively regulates intratumoral androgen synthesis in breast cancer tissues. DHT concentrations in breast cancer tissues increase significantly after neoadjuvant AI therapy compared to levels in untreated tissues [15], indicating that intratumoral DHT synthesis and the expression of androgen-responsive genes are also upregulated following AI treatment [15,27].

### 2.2. ER and AR Signaling

The nuclear ERs, which include ERα and ERβ, are part of the nuclear receptor superfamily and consist of three functional domains: the transactivation domain, DNA-binding domain, and ligand-binding domain [28]. Activation of nuclear steroid receptors begins when ligands bind to the C-terminal ligand-binding domain, prompting the receptor to translocate to the nucleus. Once in the nucleus, these receptors act as transcription factors by binding to enhancer regions such as the estrogen-responsive element (ERE) [28]. Signaling through ERs can initiate both long-term genomic signaling and rapid, nongenomic signal transduction through ligand-dependent and ligand-independent pathways, respectively [28]. In general, in ERα/β-positive breast cancers, ERα promotes tumor cell proliferation, while ERβ inhibits ERα-mediated transcriptional activity, ultimately suppressing cell growth [29]. In addition to ERα, AR is expressed in 70–90% of breast cancers. Similar to ERs, AR belongs to the nuclear receptor superfamily, and its activation begins when a ligand binds to it, prompting AR to move to the nucleus, where it acts as a transcription factor by binding to the androgen-responsive element (ARE) [30]. While androgens were initially thought to suppress breast cancer growth [31,32], increasing evidence suggests they also play a role in promoting tumor development in both ERα-positive and ERα-negative breast cancers [30,33].

## 3. Stomal Cells and Estrogen/Androgen Signaling in Breast Cancer Cells

### 3.1. The Altered Profile of Stromal Cells Based on ER/AR Status in Breast Cancer Tissues

**Figure 1:**
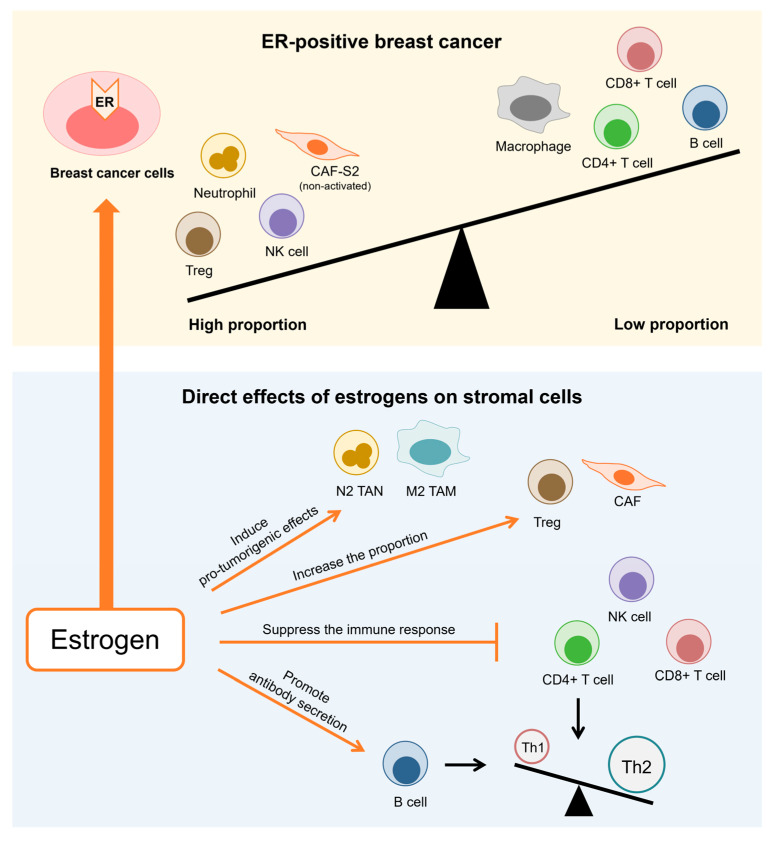
Estrogen receptor (ER)+ breast cancers are typically considered immunologically cold, characterized by an immunosuppressive tumor microenvironment with relatively low levels of tumor-infiltrating lymphocytes. ER expression in breast cancer cells is inversely correlated with lymphocyte infiltration within the tumor, particularly CD4+ T cells, CD8+ T cells, B cells, and macrophages. Conversely, ER+ breast cancers generally exhibit a higher presence of regulatory T cells (Tregs), natural killer (NK) cells, cancer-associated fibroblast (CAFs), which are less activated, and tumor-associated neutrophils (TAN). Additionally, estrogens dampen immune activity and enhance pro-tumorigenic effects through ER signaling in stromal cells. Notably, estrogens contribute to shifting the immune balance from a Th1 to a Th2 response. TAM, tumor-associated macrophage.

ER+ breast cancers are regarded as immunologically cold, featuring a tumor microenvironment that is predominantly immunosuppressive and has relatively few tumor-infiltrating lymphocytes (Figure 1). The majority of patients with ER+ breast cancer show limited responses to immune checkpoint inhibition [34]. Previous studies have identified a negative correlation between ER expression in breast carcinoma cells and the intratumoral infiltration of lymphocytes, notably CD4+ T cells, CD8+ T cells, B cells, and tumor-associated macrophages (TAMs). In contrast, ER+ breast cancers tend to have a higher prevalence of regulatory T cells (Tregs), natural killer (NK) cells, and neutrophils [13,35,36,37,38,39,40,41,42,43]. Recently, Hanamura et al. investigated the relationship between the expression of ER, progesterone receptor (PR), and AR, along with the immunological profile in breast cancer, utilizing bioinformatics analysis and flow cytometry [13]. This study revealed that ER expression is linked to a reduction in tumor-infiltrating lymphocytes (TILs), especially CD4+ T cells. We previously demonstrated that infiltration of CD163-positive M2 macrophages was negatively correlated with ER status in 116 breast carcinoma tissues, as shown by immunohistochemistry [43]. In addition, in ER+ breast cancer, the most common type of cancer-associated fibroblasts (CAFs) is CAF-S2, which is less activated and promotes less inflammation than other CAF subsets [44]. A transcriptomic study of dendritic cell (DC) subsets across different breast cancer subtypes demonstrated that dendritic cells in ER+ cancers exhibit a less activated type 1 interferon (IFN) pathway compared to those in triple-negative breast cancer (TNBC) [45]. Furthermore, an increase in TILs during letrozole treatment (AI therapy) was significantly correlated with a poor therapeutic response in ER+ breast cancer patients. This elevation in TILs during endocrine therapy may reflect immunogenicity, indicating that these patients might be suitable candidates for immunotherapy [46].

**Figure 2:**
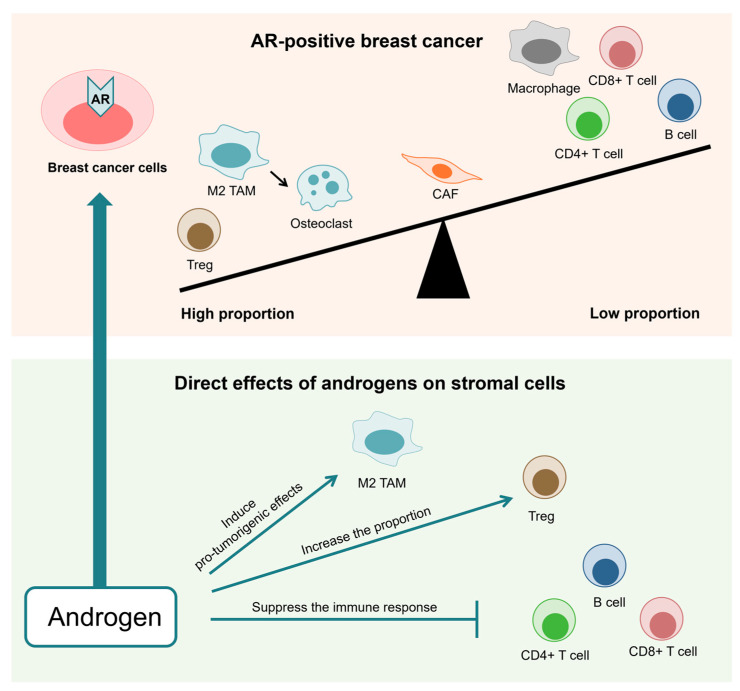
Androgen receptor (AR) expression in breast cancer cells is inversely associated with immune cell infiltration and cytotoxic immune activity, suggesting an immunosuppressive role of AR signaling in breast cancer. High AR expression is correlated with increased regulatory T cells (Tregs), while low AR expression is associated with greater infiltration of immune cells such as CD4+ T cells, CD8+ T cells, and B cells. Additionally, androgens directly enhance immunosuppressive effects and promote pro-tumorigenic functions in stromal cells.

The expression of the AR in breast cancer cells, like that of the ER, is inversely correlated with immune cell infiltration and cytotoxic immune activity, indicating that AR signaling has an immunosuppressive effect in breast cancer [13,47] (Figure 2). An analysis of the relationship between AR expression and the immunological profile in breast cancer using immunohistochemistry, flow cytometry, and bioinformatics revealed that high AR expression is associated with Tregs, whereas low AR expression is linked to various immune cells, including CD4+ T cells, CD8+ T cells, gamma delta T cells, memory B cells, and TAMs [13,48,49,50].

### 3.2. ERE/ARE and Regulation of Immunological Profiles in Breast Cancer

These regulatory mechanisms can occur either directly via EREs/AREs or through interactions between ER/AR and other transcription factors, such as NF-κB. Interestingly, previous studies have demonstrated that EREs are present in the promoters of Fas ligand (FasL) and that estrogens promote FasL expression in ER+ breast cancers [51,52]. Fas-mediated apoptosis of TILs is triggered by interaction with FasL, and recent evidence suggests that tumor cells expressing FasL may use the Fas-FasL system to induce TIL apoptosis, potentially evading immune surveillance [53]. On the other hand, multiple studies have shown that E2 can either promote or suppress the secretion of IFN-γ, which is essential for stimulating the antitumor immune response, likely through the direct interaction of ER with EREs in the promoter region of the IFN-γ gene [54,55,56]. Interleukin (IL)-6, which acts as either a pro-inflammatory cytokine or an anti-inflammatory cytokine, has been identified as an AR-responsive gene, with its expression increased via the ARE [57]. Furthermore, it has been reported that AREs exist in the promoter region of the transforming growth factor (TGF)-β gene, an anti-inflammatory cytokine, and that they positively or negatively regulate the expression of TGF-β [58]. Thus, the regulation of immune-related factors via EREs and AREs is diverse, suggesting that ER/AR signaling in breast cancer cells likely exerts a complex control over stromal cells.

### 3.3. Mechanism of Stomal Cell Regulation by ER/AR Signaling in Breast Cancer Cells

E2/ERα signaling has been shown to influence the regulation of human leukocyte antigen class II (HLA-II) in breast cancer cells based on in vitro studies [59]. Normally, HLA-II expression is limited to professional antigen-presenting cells. However, recent studies have found that some cancer cells also express HLA-II in an IFN-γ-inducible manner, and the presentation of HLA-II neoantigens by cancer cells to CD4+ T cells plays an important role in activating tumor immunity [59,60]. In the TNBC cell line MDA-MB-231, which overexpress wild-type ERα, the level of HLA-II is significantly lower compared to control cells, and this reduction is further intensified by E2 treatment. Recent single-cell analyses of T cell populations across various breast cancer subtypes provide additional insights, revealing that ER+ breast cancers have a lower diversity in the T cell receptor repertoire, which aligns with the limited presence of neoantigens in ER+ cancer when compared to TNBC and HER2+ cancers [61,62].

Current findings on the effects of ER− and ER+ breast cancer cells in regulating macrophage infiltration and function remain inconsistent. In vitro experiments using cell lines show that the ability of the non-TNBC cell line to promote macrophage migration is weaker compared to the TNBC cell lines [63]. Conversely, estrogen-stimulated production of chemokines CCL2 and CCL5 by ER+ breast cancer cells has been linked to the recruitment of TAMs, which subsequently promotes cancer cell dissemination [64,65]. Regarding the regulation of macrophage function, analysis of tumors from the TCGA and METABRIC databases suggests that ER+ cancers are more likely to express an M2 macrophage signature, while TNBCs exhibit higher M1 polarization [66], a finding supported by single-cell RNA sequencing analyses [67]. However, macrophages co-cultured with TNBC cells showed significantly higher expression of M2 phenotype markers compared to those co-cultured with ER+ breast cancer cells [68]. The same research group investigated macrophage activation in response to co-culture with various breast cancer cell lines and found that high granulocyte colony-stimulating factor (G-CSF) secretion by the TNBC cell line resulted in immunosuppressive macrophages, which promoted cancer cell migration [69]. However, these co-culture experiments were conducted in the absence of estrogen, so the observed effects may be attributed to inherent differences in the properties of the cell lines, independent of estrogen.

Long non-coding RNAs (lncRNAs) are RNA molecules longer than 200 nucleotides that do not code for proteins. These lncRNAs can modify chromatin through lncRNA–protein interactions or lncRNA–DNA interactions, thereby regulating various processes [70]. Recently, Huang et al. developed a risk model by analyzing nine lncRNAs associated with androgen receptor signaling pathways (ARSP-related lncRNAs) to predict the prognoses of breast cancer patients [70]. The results indicated that this risk model, based on ARSP-related lncRNAs, was strongly linked to different immune cell clusters and immune cell infiltration.

Previous studies revealed that AR+ breast cancer tissues are less infiltrated with macrophages. On the other hand, a recent study revealed that AR interactions with c-Myc in breast cancer cells mediate the M2 polarization of macrophages [71]. Additionally, this interaction encourages the differentiation of M2 macrophages into osteoclasts and enhances osteoclast activity, leading to increased bone resorption and promoting bone metastasis in luminal androgen receptor-type (LAR)-TNBC [71]. Analysis of publicly available clinical databases shows that LAR-TNBC has a pronounced tendency for bone metastasis. Preclinical data also suggest that androgens promote tumorigenesis in ERα-negative breast cancers, particularly in TNBC, aligning with the increased androgen activity seen in LAR-TNBC subtypes [72,73]. Regulation of TAMs may be one factor contributing to the tumor-promoting effects of androgens in the LAR-TNBC subtype.

Co-culturing CAFs with the MDA-MB-453 LAR TNBC cell line increases the expression of androgen synthesis enzymes 17βHSD2, 17βHSD5, and 5α-Red1 in breast cancer cells [74]. Furthermore, immunohistochemistry indicates that the status of α-SMA, a marker of CAFs, is significantly correlated with the expression of 17βHSD2 and 17βHSD5 in TNBC tissues, particularly in AR-positive cases [74]. These findings indicate that CAFs both upregulate androgen synthesis enzymes in breast cancer cells and are potentially influenced by AR signaling within these cells.

## 4. Estrogen and Androgen Signaling in Stromal Cells of Breast Cancer

### 4.1. TILs and Estrogens

Both ERα and ERβ are expressed in T cells. In general, CD4+ T cells exhibit higher levels of ERα than ERβ, whereas CD8+ T cells show comparable levels of both receptors [75,76,77]. E2 regulates the Th1/Th2 balance. It has been reported that E2 can suppress the production of Th1 pro-inflammatory cytokines, such as IFNγ, IL-2, IL-12, and tumor necrosis factor (TNF) α [78,79,80]. On the other hand, E2’s effects on Th2 cells include increasing anti-inflammatory cytokines, such as IL-10, IL-4, and TGF-β, with the rise in IL-4 linked to increased expression of the key Th2 transcription factor GATA-3 [79,81,82,83,84]. The balance between Th1 and Th2 cells is critical for antitumor immunity. Th1 cells are known for their potent anticancer effect, and a shift towards a Th2-dominant response during the perioperative period is positively correlated with poor prognosis in breast cancer patients [85,86]. Therefore, estrogen may contribute to the suppression of tumor immunity by promoting a Th1 to Th2 shift in immune balance.

In terms of receptor function, studies in mouse models of inflammatory diseases have shown that ERα and ERβ signaling often have opposing roles in CD4+ T cells, with ERα promoting inflammation and ERβ acting as an anti-inflammatory mediator [14,87]. However, in a mouse model of mammary cancer, mutations in ERβ resulted in reduced infiltration of CD4+ and CD8+ T cells and lower levels of IFNγ within the tumor microenvironment, leading to tumor growth [88]. In lung and cervical tumor samples, ERα signaling has been linked to decreased infiltration of CD4+ and CD8+ T cells into the tumor microenvironment [89,90]. These findings suggest that the roles of ERα and ERβ in immune responses may vary depending on the context of the disease and species differences.

Estrogens/ER upregulate Foxp3 expression in Tregs by directly binding to EREs, thereby inducing immunosuppressive properties [89,91,92]. In human breast cancer, patients treated with letrozole alone or in combination with an antineoplastic agent (cyclophosphamide) showed a significant reduction in Treg numbers [93,94]. Additionally, another in vivo study demonstrated that ICI 182780, an ERα and ERβ antagonist, could reverse estradiol’s effects on inducing the Treg phenotype [95]. These effects likely encompass both the estrogen actions on breast cancer cells and the direct influence of estrogen on stromal cells. In both humans and mice, ERα and ERβ are expressed in B cells [96]. ER signaling also controls the expression and activity of activation-induced deaminase (AID), a critical enzyme for antibody production, through interaction with EREs in the promoter region of the human AID gene [97]. Treatment with E2 increases total IgG antibody production in peripheral blood and splenic B cells [98,99]. These results suggest that estrogen not only enhances the activity of CD4+ Th2 cells but also promotes a Th2-dominant immune environment by directly affecting B cells. NK cells also express both ERα and ERβ [100], and the impact of ER signaling in NK cells on various malignancy outcomes has been studied [101,102,103]. High concentrations of E2 and signaling through ERα reduce NK-cell-induced death in breast cancer cell lines by increasing granzyme B inhibitors and proteinase inhibitor 9, whereas treatment of NK cells with tamoxifen impairs cytotoxicity [102].

### 4.2. TAMs and Estrogens

While earlier studies reported that monocytes express ERβ and macrophages express ERα [104,105], Harkonen et al. demonstrated that both receptors are present in macrophages [106]. Additionally, the G-protein-coupled estrogen receptor (GPER), a third type of ER, is a seven-transmembrane receptor belonging to a superfamily known for modulating kinase pathways and second messengers, and it has been frequently identified in macrophages [107]. On the other hand, the expression of ER in TAMs within tumor tissues remains unclear. Estrogen treatment modulates various macrophage functions, including the production of anti-inflammatory factors and antioxidant enzymes, lipid metabolism, and phagocytic activity, thereby influencing both the innate and adaptive immune systems [39]. It is widely recognized that estrogen/ER signaling drives macrophages toward an anti-inflammatory state. ERα and GPER play a key role in suppressing the secretion of pro-inflammatory markers, such as TNF-α and IL-8, associated with M1 macrophages [108,109,110,111]. Toniolo et al. showed that pre-treatment with E2 reverses the LPS/IFNγ-induced downregulation of M2 markers [110]. Thus, estrogen/ER signaling in TAMs may contribute to their pro-tumorigenic and immunosuppressive roles within the tumor microenvironment, warranting further study using breast carcinoma tissues.

On the other hand, estrogen may also exert antitumor effects through macrophages. E2 treatment promotes M1-like macrophage activation through cadherin-11 to aggravate temporomandibular joint inflammation in rats [112]. E2 also stimulated nitric oxide release in human peripheral monocytes and a murine macrophage cell line via GPER activation [113]. Estrogens also promote the release of arachidonic acid and the production of prostaglandin E2 (a derivative of arachidonic acid) in human monocytic cell lines, boosting inflammatory responses [114]. Further studies are needed to determine the impact of estrogen on macrophages in relation to breast cancer progression.

### 4.3. CAFs and Estrogens

Although CAFs within breast carcinoma tissues express ER [115], limited studies have examined the effects of estrogen on CAFs in breast cancer. ERα36, an alternatively spliced variant of ERα, is identified as a pro-tumorigenic factor in carcinoma cells. Moreno et al. demonstrated that CAFs in breast carcinoma tissues express ERα36, and a high presence of ERα36-positive CAFs is linked to the invasive phenotype of TNBC cells, potentially leading to a poor prognosis for TNBC patients [116]. Notably, GPER is frequently reported to be expressed in breast CAFs, where it regulates their function [117,118,119]. The activation of GPER in CAFs influences hypoxia-driven invasion of breast cancer cells in a connective tissue growth factor-dependent manner [119]. E2, tamoxifen, and G1, an agonist of GPER, enhance CAF proliferation and cell cycle progression through GPER activation [118]. Madeo et al. found that GPER stimulates the expression of c-FOS, cyclin D1, and connective tissue growth factor in breast CAFs in response to E2, thereby promoting CAF proliferation [117]. Additionally, tamoxifen and G1 boost E2 production in breast CAFs via GPER/EGFR/ERK signaling when testosterone, the substrate for estrogen, is added to the medium [118].

### 4.4. Other Types of Cells and Estrogens

Neutrophils are essential immune cells that act as the first line of defense against pathogens and can adopt either an anti-tumorigenic N1 phenotype or a pro-tumorigenic N2 phenotype [11,120]. In a mammary involution mouse model, mice were administered E2 and injected with a 4T1 TNBC cell line, resulting in E2-induced infiltration of neutrophils in the mammary tissue [121]. Furthermore, neutrophil depletion using antibodies led to a marked reduction in estrogen-induced mammary tumor growth [121]. In other in vitro and in vivo breast cancer studies, E2 promoted N2-neutrophil polarization and was correlated with tumor progression and metastasis [122]. Taken together, the activity of mammary neutrophils is significantly regulated by E2 and plays an important role in cancer progression.

DCs are the potent antigen-presenting cells, and following their antigen stimulation, DCs secrete pro-inflammatory cytokines to stimulate T lymphocytes and initiate immune responses [123]. DCs and progenitor subsets express both ERα and ERβ, and previous studies illustrate that estrogen/ER signaling is essential for regulating differentiation, cytokine production, and activity of DCs [124,125]. However, to date, few studies have characterized the functional role of ER signaling in DCs in the context of breast cancer outcomes.

### 4.5. TILs and Androgens

AR expression is also observed in immune cells, including T and B cells, macrophages, monocytes, and neutrophils, suggesting that androgens play a role in regulating immune responses [126]. Sex-related differences in immunotherapy responses have been documented, with previous studies highlighting the immunosuppressive effects of androgens [127,128,129]. Recently, Zhang et al. found that androgen/AR signaling dampens T-cell-mediated immunity in males by upregulating ubiquitin-specific protease 18 expression, which in turn inhibits TGFβ-activated kinase 1 phosphorylation and the activation of NF-κB in anti-tumor T cells [130]. Additionally, AR suppresses the activity and stemness of male tumor-infiltrating CD8+ T cells by modulating epigenetic and transcriptional programs, such as androgen-driven IFNγ repression [131,132]. Androgens also inhibit antibody production by suppressing B-cell lymphopoiesis and germinal center formation [133]. In addition, androgens influence the expansion and function of Tregs by promoting FOXP3 expression through ARE [134].

The immunosuppressive effect of androgens on lymphocytes, as described above, may contribute to sex-related differences in tumor incidence and outcomes, suggesting a potential therapeutic target for cancers [135]. Clinical trials show that androgen deprivation therapy (ADT), a common treatment for prostate cancer, can promote the expansion of naïve T cells and enhance T-cell responses [136]. Guan et al. demonstrated that blocking AR increased sensitivity to PD-1-targeted immune checkpoint inhibitors by directly improving CD8+ T cell function in metastatic castration-resistant prostate cancer [131]. In human colorectal cancer and skin cutaneous melanoma, AR expression in CD8+ T cells was associated with their exhaustion [132]. Disruption of the androgen–AR axis, either through surgical castration or with abiraterone, significantly enhanced T cell anti-tumor activity in male mice and improved the efficacy of anti-PD-1 immunotherapy in bladder cancer, colorectal cancer, and melanoma [130,132,137]. Although the role of androgen/AR signaling in lymphocytes in breast cancer is not yet well understood, recent research by Li et al. investigated the relationship between immune checkpoint receptor expression on T cells in breast cancer tissues and androgen levels in the serum of breast cancer patients [138]. They discovered that testosterone and DHT levels were positively correlated with PD-1 expression on Vδ1+ T cells in patients with HER2-positive and luminal B breast cancers. These findings suggest a potential therapeutic approach of combining androgens with PD-1 blockade for treating HER2-positive and luminal B breast cancers.

### 4.6. TAMs and Androgens

Recent research has shown that macrophages express AR and play key roles in various human diseases, including breast cancer [43,139]. This suggests that intratumoral androgens may influence not only breast carcinoma cells but also TAMs. In our previous study, we demonstrated AR expression in TAMs within breast carcinoma tissues, and a phenotype characterized by 5αRed1-positive and higher macrophage infiltration was strongly associated with more aggressive tumor behavior and poorer clinical outcomes in breast cancer patients [43]. Experiments using 4T1 murine breast cancer cells and RAW264.7 macrophages showed that androgens amplify the pro-tumorigenic activities of macrophages, enhancing both in vitro cell proliferation and in vivo tumorigenesis. However, although the androgen/AR signaling pathway in TAMs is crucial for prostate cancer progression [140,141], its direct role in regulating TAMs in breast cancer remains largely unexplored.

### 4.7. CAFs and Androgens

AR expression in CAFs within breast carcinoma tissues has also been confirmed through RT-PCR and immunohistochemistry using CAFs isolated from primary breast tumor biopsies [142]. In breast cancer, androgens are thought to inhibit the motility of MCF-7 breast cancer epithelial cells by promoting the secretion of soluble factors via AR signaling activation in CAFs [142]. However, the specific role of androgens in CAFs in the context of breast cancer remains unclear. In an orthotopic skin cancer model, AR loss in human dermal fibroblasts increased the tumorigenic potential of squamous cell carcinomas and melanoma cells [143]. More recently, the same research group demonstrated that AR loss in CAFs led to the phosphorylation of lamin A/C, key structural components of the nuclear scaffold, which in turn regulated the expression of myofibroblast subtype CAF effector genes [144]. Exploring how AR function in CAFs influences breast cancer progression may offer new insights into the role of CAFs in breast cancer.

## 5. Conclusions

**Figure 3:**
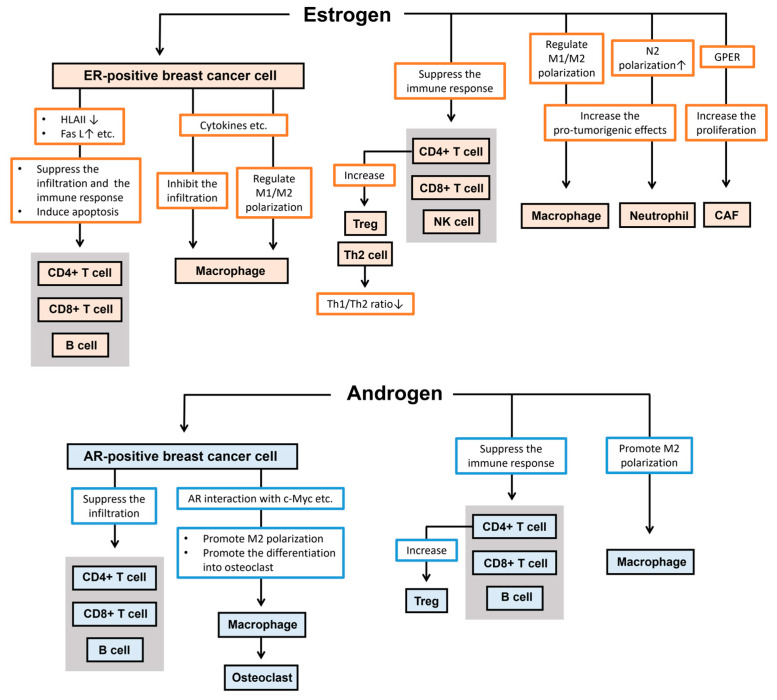
Estrogens and androgens regulate the breast cancer microenvironment through diverse mechanisms. Overall, estrogen/ER and androgen/AR signaling in breast cancer cells or stromal cells generally contribute to the promotion of an immunosuppressive tumor microenvironment. AR, androgen receptor; CAF, cancer-associated fibroblast; ER, estrogen receptor; FAS L, fas ligand; GPER, G-protein-coupled estrogen receptor; HLAII, human leukocyte antigen class II; NK, natural killer; TAM, tumor-associated macrophage; Th, T helper cell; Treg, regulatory T cell.

Sex steroid hormones regulate the breast cancer microenvironment through various mechanisms (Figure 3). Overall, estrogen/ER signaling and androgen/AR actions in breast cancer cells tend to promote an immunosuppressive tumor microenvironment. Although estrogens and androgens have frequently been reported to induce direct immunosuppressive properties in stromal cells, there are still few studies demonstrating the impact of these effects on breast cancer progression, and further research is needed to clarify this in the future.

## Data Availability

No new data were created or analyzed in this study. Data sharing is not applicable to this article.
